# Systemic and Mucosal Immune Responses From Serotype‐Switched Viral‐Vectored Ebola Vaccines

**DOI:** 10.1002/jmv.71161

**Published:** 2026-09-21

**Authors:** Joshua Wiggins, Adthakorn Madapong, Eric Weaver

**Affiliations:** ^1^ Nebraska Center for Virology University of Nebraska—Lincoln Lincoln Nebraska USA; ^2^ School of Biological Sciences University of Nebraska—Lincoln Lincoln Nebraska USA

**Keywords:** adenovirus serotype, ebola, recombinant adenovirus, vaccine, viral vector

## Abstract

Ebola virus is a highly virulent pathogen causing severe hemorrhagic fever with 25%–90% mortality. The 2014 outbreak infected over 28,000 people, causing 11,325 deaths. Although there is one FDA‐approved vaccine available for limited use, additional vaccines are needed. We developed adenoviral‐vectored vaccines expressing the ebolavirus glycoprotein (GP), the sole viral surface protein and primary immunogen. Vaccine constructs spanned three species and five human adenovirus subtypes, including low‐seroprevalent serotypes to minimize pre‐existing immunity. We compared single‐dose and prime‐boost regimens, intramuscular versus needle‐free intranasal delivery, and serotype‐switching strategies. Immunogenicity was assessed by quantifying GP‐specific antibodies and T cell responses in BALB/c mice. Species C vectors (Ad5 and Ad6) elicited strong antibody responses and promoted rapid class switching to IgG compared to species B and D vectors. Serotype‐switched regimens further enhanced antibody levels via intramuscular or intranasal routes and generated robust T cell responses when delivered intramuscularly. Intranasal vaccination induced detectable IgA but no measurable T cell responses, indicating route‐dependent immune compartmentalization. Sera partially inhibited viral entry in a dose‐dependent manner, with the greatest reduction observed in the Ad5/Ad6 regimen. Collectively, adenoviral species selection, serotype switching, and route of administration significantly influence GP‐specific immunity and warrant further evaluation in protective challenge models.

## 1 Introduction

Ebola virus (EBOV) is an enveloped, negative‐sense, single‐stranded RNA virus belonging to the Filoviridae family. Six species are currently recognized: Zaire ebolavirus, Sudan ebolavirus, Reston ebolavirus, Taï Forest ebolavirus, Bundibugyo ebolavirus, and Bombali ebolavirus [1]. Among these, Zaire ebolavirus (ZEBOV) is responsible for most of the large outbreaks, including the 2014–2016 West African outbreak, and is associated with case fatality rates reaching up to 90% in some epidemics [2]. Since its initial identification in 1976, EBOV has caused recurrent outbreaks, primarily in sub‐Saharan Africa, characterized by severe hemorrhagic fever and high mortality [3, 4]. Transmission occurs through direct contact with infected bodily fluids or contaminated materials, placing healthcare workers and their close contacts at elevated risk [5]. Although considerable progress has been made in understanding EBOV pathogenesis and immunity, licensed antiviral drugs and vaccines remain limited. Currently, one U.S. FDA–approved vaccine (Ervebo) and one European Commission–approved two‐dose regimen (Zabdeno/Mvabea) are available [6]. Continued outbreaks, including recent cases in the Democratic Republic of the Congo, highlight the persistent threat posed by EBOV and the need for improved and more accessible vaccine strategies [7].

The EBOV glycoprotein (GP) is the sole viral surface protein and mediates viral entry through host cell attachment and membrane fusion [8]. As the only surface‐exposed antigen, GP is the primary target of neutralizing antibodies and a key antigen for both humoral and cellular immune responses. Vaccine platforms delivering full‐length GP or selected GP domains have demonstrated protective efficacy in both preclinical models and clinical trials, validating GP as a critical immunogen [9, 10]. However, current vaccine approaches experience practical limitations. For example, Ervebo is a live, replication‐competent vesicular stomatitis virus‐based vaccine, which may restrict its use in certain populations such as immunocompromised and pregnant individuals [11]. In addition, stringent cold‐chain requirements complicate deployment in outbreak‐prone regions with limited infrastructure. These challenges underscore the importance of developing alternative vaccine platforms that maintain strong immunogenicity while improving safety, stability, and distribution feasibility.

Adenoviruses (AdVs) represent a versatile and well‐characterized vaccine platform capable of inducing potent innate activation and robust humoral and cellular immune responses [12]. Recombinant AdV vectors can be engineered to express EBOV GP, resulting in robust antigen expression and efficient immune priming [13, 14]. Adenoviral vectors have demonstrated safety and efficacy in multiple licensed and investigational vaccines, including their use in the Zabdeno component of the Zabdeno/Mvabea regimen [15, 16], as well as in vaccines targeting other emerging infectious diseases [17, 18, 19]. Importantly, AdV‐based vaccines can be manufactured at scale and adapted rapidly in response to evolving outbreaks [20]. Building on previous work in our laboratory characterizing AdV‐vectored vaccines against HIV, influenza A and B, and Zika virus [21, 22, 23, 24], we evaluated five recombinant AdV vectors expressing the Ebola virus Mayinga GP. We compared routes of administration to assess the induction of systemic versus mucosal immunity and investigated adenoviral serotype switching strategies to enhance the magnitude and breadth of immune responses. Together, these studies define how adenoviral species selection, serotype switching, and vaccination route influence the magnitude and quality of EBOV‐specific immune responses.

## 2 Materials and Methods

### 2.1 Ethics

All experimental procedures conducted in this study were approved by the University of Nebraska‐Lincoln Institutional Biosafety Committee (Protocol #619). Additionally, all experiments involving mice were approved by the Institutional Animal Care and Use Committee (Protocol #2704) and were conducted in accordance with institutional and federal guidelines for the care and use of laboratory animals. Four‐ to six‐week‐old, female BALB/c mice were purchased from Jackson Laboratories. Animals were housed in a temperature‐controlled facility with five mice per cage and maintained on a 14‐h light, 10‐h dark cycle. Food and water were provided ad libitum.

### 2.2 Cells

Human Embryonic Kidney 293 (HEK293) and 293‐E4‐pIX cells were obtained from American Type Culture Collection (ATCC, Manassas, VA, USA) and Microbix Biosystems, respectively. TZM‐bl reporter cells were obtained from the Biodefense and Emerging Infectious (BEI) Resource Repository (HRP‐8129). Cells were maintained in a humidified incubator at 37°C and 5% CO_2_ and cultured in Dulbecco's Modified Eagle's Medium (DMEM, Gibco) supplemented with 10% fetal bovine serum and 1% penicillin/streptomycin (complete DMEM).

### 2.3 Vaccine Production

The Zaire Ebolavirus (Mayinga isolate) glycoprotein (GP) gene (GenBank accession: NP_066246.1) was codon‐optimized for humans and synthesized by GenScript into the pUC57 plasmid. The GP gene, under the control of the cytomegalovirus (CMV) immediate‐early promoter and SV40 polyadenylation signal, was cloned into adenoviral shuttle plasmids for each of the recombinant Adenoviruses as previously described [25, 26]. Linearized shuttle plasmids were co‐transformed with Adenoviral genomic DNA in *E. coli* BJ5183 cells to facilitate homologous recombination, and the recombinant adenovirus plasmids were subsequently amplified in *E. coli* XL‐1. Recombinant plasmids were linearized with *PacI* and transfected into HEK293 (Ad5 and Ad6) or 293‐E4‐PIX (Ad7, Ad28, and Ad48) cells using PolyFect (Qiagen 301105). Rescued viruses were amplified through serial passages in HEK293 or 293‐E4‐pIX cells, culminating in a Corning 10‐cell stack to produce a high viral titer. Recombinant adenoviruses were purified through two rounds of Cesium Chloride density‐gradient ultracentrifugation, followed by desalting using Econo‐Pac 10DG columns (Bio‐Rad). Viral particles were quantified by spectrophotometry absorbance at 260 nm, split into aliquots, and frozen at −80°C for long‐term storage as previously described [27]. Viral DNA was purified by following the manufacturer's instructions in a PureLink Viral RNA/DNA Mini Kit. DNA sequencing was performed using an Oxford Nanopore MinION Mk1B and Native Barcoding Kit (SQK‐NBD114.24). Sequence assembly was performed using the Epi2Me Amplicon workflow (v5.3.1).

### 2.4 Western Blotting

To confirm Ebola GP expression from the recombinant Ad vectors, Western blotting was performed as previously described [23]. Briefly, HEK293 cells were cultured in a 12‐well plate until confluent and then infected at an MOI of 100 for 48 h. The cells were harvested, resuspended in 2× Laemmli buffer (BioRad) with beta‐mercaptoethanol, and lysed at 95°C for 10 min. Lysates were resolved on a 12% SDS‐PAGE gel and then transferred to a nitrocellulose membrane. The membrane was blocked in tris‐buffered saline with 0.1% Tween‐20 (TBST) containing 5% skim milk for 2 h. GP expression was detected using an HRP‐conjugated rabbit anti‐Zaire ebolavirus GP polyclonal antibody (CSB‐PA314099LB01ZAT) diluted 1:2500 in TBST containing 2% milk. After overnight incubation at 4°C, the membrane was washed five times with TBST, developed for 10 min using SuperSignal West Pico Chemiluminescent Substrate (Thermo Scientific), and developed on a ChemiDoc MP imaging system (BioRad).

### 2.5 Vaccinations

Mice were immunized on Day 0 with a total of 1 × 10^10^ viral particles (vp) administered either intramuscularly (im) or intranasally (IN). For im vaccination, mice were anesthetized with isoflurane and vaccinated with 25 µL injected into each hind leg muscle (50 µL total volume). For IN vaccination, mice were anesthetized with a mixture of ketamine/xylazine delivered intraperitoneally, followed by a 10 µL dose of vaccine pipetted into each nostril (20 µL total volume) [28]. Sterile DPBS was used as a mock vaccine for each delivery route. On Day 21, mice were bled via the submandibular vein to assess immune responses following prime immunization. Mice received either a homologous or heterologous boost vaccine on Day 21 using the same dose and route. Mice were sacrificed on Day 31 for blood and spleen collection. Whole blood was collected in BD Microtainer SST collection tubes (Becton‐Dickinson 365967) and spun at 6000*g* for 5 min to separate serum.

### 2.6 Enzyme‐Linked Immunosorbent Assay (ELISA)

High‐binding 96‐well plates were coated overnight at 4°C with 1:100 diluted, irradiated Ebola virus cell lysate (BEI, NR‐49809), as this lysate contains full‐length glycoprotein in its native conformation. Plates were washed three times with DPBS containing 0.01% Tween‐20 (DPBS‐T) and blocked for 2 h with DPBS‐T containing 4% bovine serum albumin (BSA). Serum samples were heat‐inactivated at 56°C for 30 min and diluted 1:100 in DPBS‐T containing 2% BSA. Fifty microliters of diluted serum were added per well and incubated for 1 h at room temperature. Plates were then washed with DPBS‐T and incubated for 1 h with 50 µL/well of secondary antibody diluted 1:5000 in DPBS‐T containing 2% BSA. Secondary antibodies used were goat anti‐mouse IgG (H + L) HRP (Sigma AP308P), goat anti‐mouse IgA HRP (Southern Biotech 1040‐05), and goat anti‐mouse IgM HRP (Southern Biotech 1021‐05). After additional washing with DPBS‐T, plates were developed with 50 µL of 1‐shot TMB Ultra ELISA substrate for between 5 and 15 min, then stopped with 50 µL 2 M sulfuric acid when noticeable color developed. Absorbance was measured at 450 nm on a SpectraMax i3x plate reader. Background absorbance was subtracted from each well to determine the antigen‐specific antibody responses.

### 2.7 IFN‐γ Enzyme‐Linked Immunospot (ELISpot) Assay

Splenocytes were isolated from harvested spleens by mechanical disruption through a 40 µm Nylon Mesh cell strainer (Fisher 50‐136‐7927), followed by red blood cell lysis using 5 mL ACK buffer. The ACK reaction was stopped with 5 mL cold DPBS, followed by centrifugation at 300*g* for 5 min. Cells were washed and resuspended in RPMI media + 5% FBS (complete RPMI) and counted using a TC‐20 automated cell counter (BioRad). Multiscreen Immobilon‐P hydrophobic 96‐well plates (Millipore S2EM004M99) were coated overnight at 4°C with 250 ng of anti‐mouse IFN‐γ mAb (Mabtech AN18) and blocked for 3 h at 37°C with complete RPMI. Splenocytes were adjusted to a concentration of 2500 viable cells/µL and added to appropriate wells at a volume of 100 µL/well. Next, a peptide library containing 17‐mer peptides with 11 amino acid overlaps, spanning the EBOV Mayinga GP was added to each well at a concentration of 200 ng/peptide/well suspended in 50 µL of complete RPMI. Concanavalin A (Sigma C0412‐5MG) and complete RPMI were used in place of peptides to serve as positive and negative controls, respectively. After overnight incubation at 37°C with 5% CO_2_, plates were washed six times with DPBS‐T and treated with a biotinylated anti‐mouse IFN‐γ detection mAb (Mabtech R4‐6A2) for 1 h at room temp, followed by streptavidin‐alkaline phosphatase (Mabtech 3310‐10‐1000) for 1 h. Spots were developed with room temperature BCIP/NBT substrate (Mossbio NBTH‐500) for 5 min, followed by washing thoroughly with ddH_2_O and air‐drying overnight. Spots were counted using a CTL ImmunoSpot reader (Cellular Technologies, USA). Background spot counts from unstimulated wells were subtracted, and results were expressed as spot‐forming units (SFU) per 10^6^ splenocytes.

### 2.8 Ebola Pseudovirus Production

Non‐infectious Ebola pseudoviruses were produced as previously described using a two‐plasmid system [29]. The HIV‐1 backbone plasmid pSG3ΔEnv (BEI, ARP‐11051) and pcDNA3.1_EbolaGP (BEI, NR‐19814) envelope plasmid were co‐transfected into HEK293 cells cultured in complete DMEM at ~70% confluency. Plasmids were co‐transfected at a ratio of 3:1 backbone to envelope using PolyFect (Qiagen) as the transfection reagent. Supernatants were harvested 72 h post‐transfection, clarified by centrifugation at 300*g* to remove cellular debris, split into single‐use aliquots, and stored at −80°C until use.

### 2.9 Pseudovirus Neutralization Assay

Neutralization activity of vaccine‐induced sera was evaluated using Ebola pseudoviruses and a luciferase reporter cell line as previously described [29]. Serum samples were heat‐inactivated at 56°C for 30 min and diluted 1:50 in complete DMEM, followed by additional fivefold serial dilutions. TZM‐bl reporter cells were cultured overnight in complete DMEM on black, 96‐well plates (Corning 3603) at a density of 10,000 cells/well. Cells were washed once with DPBS, and then 50 µL of diluted serum was transferred to each well. EBOV GP pseudoviruses were thawed, diluted to a final volume of 50 µL/well in DMEM, and added to cells. Plates were incubated at 37°C and 5% CO_2_ for 72 h. Cells were washed once with DPBS and then lysed in 20 µL of 1× passive lysis buffer (Promega E1910) for 15 min with gentle rocking. Relative light units (RLU) for each well were measured on a Veritas luminometer by applying 100 µL of luciferase assay buffer (Promega E1500). Virus‐only wells (100% infection) and uninfected wells were included on each plate in triplicate to establish normalization controls and verify assay functionality. Percentage of infection was calculated by first subtracting the uninfected background RLU from each well, then dividing by the virus‐only controls.

### 2.10 Statistical Analysis

All statistical analysis was performed using GraphPad Prism version 10.6.1. Comparisons among multiple groups were conducted using ordinary one‐way analysis of variance (ANOVA) followed by Dunnett's multiple comparisons test. Data are represented as mean plus or minus standard error (SEM) unless otherwise specified. A *p* value of < 0.05 was considered statistically significant.

## 3 Results

### 3.1 Vaccine Design and Production

**Figure 1:**
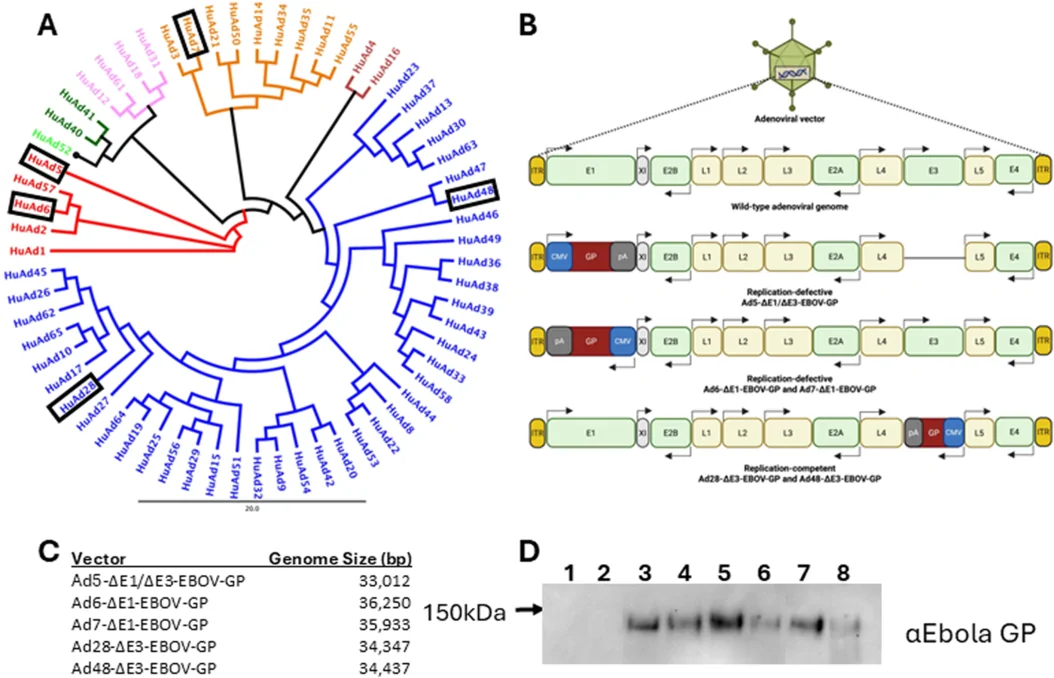
Vaccine design and protein expression. (A) Circular neighbor‐joining tree illustrating phylogenetic diversity of human Adenovirus (HuAd) hexon genes (the primary structural and immunogenic proteins). The five vectors used in this study are indicated by black boxes. Colors represent the seven different species of HuAds. Species A: Pink, Species B: Orange, Species C: Red, Species D: Blue, Species E: Brown, Species F: Green, Species G: Bright green. (B) Depiction of Adenoviral vectors showing viral inverted terminal repeats (ITR), early (E), and late (L) genes, and insertion of CMV_EBOV‐GP_pA immunogen to create recombinant vaccine vectors. Vectors were confirmed by whole genome sequencing of the viral DNA. Directionality of the genes and immunogen is indicated by arrows. (C) Table listing the vector names and total genome sizes. (D) Western blot showing GP expression in infected HEK293 cells. 1: Uninfected cells, 2: Ad26ΔE1_GFP, 3: Ad5ΔE1/ΔE3_EBOV, 4: Ad6ΔE1_EBOV, 5: Ad7ΔE1_EBOV, 6: Ad28ΔE3_EBOV, 7: Ad48ΔE3_EBOV, 8: Positive control lysate (BEI, NR‐49809). An image of the entire blot is shown in Figure S1.

To evaluate how adenoviral vectored vaccines influence EBOV immunogenicity, we generated five recombinant vectors expressing the Zaire ebolavirus (Mayinga isolate) glycoprotein (GP). The vectors spanned species B, C, and D of the human Adenoviral phylogenetic tree, shown in orange, red, and blue, respectively (Figure 1A). The GP transgene was cloned under the control of the CMV immediate early promotor and SV40 poly‐adenylation transcription terminator signal with a total size of 2857 bp. Whole‐genome sequencing confirmed the correct insertion of GP into each of the viral genomes. Figure 1B shows the layout of each viral genome, including the transgene insertion and whether the vector was replication deficient or replication competent. The total size of the genomes, including the transgene ranged from 33.0 to 36.3 kbp and is listed in Figure 1C. To confirm transgene expression, HEK293 cells were infected with each recombinant vector and GP production was evaluated by Western blot. A band corresponding to the expected molecular weight of EBOV GP (~130 kDa) was detected for all constructs, confirming successful expression (Figures 1D and S1). Together, these data validate the successful generation and characterization of the five adenoviral EBOV vaccine candidates for subsequent immunogenicity studies.

### 3.2 Species C Vectors Induce Systemic IgG Antibodies Following Prime Immunization

**Figure 2:**
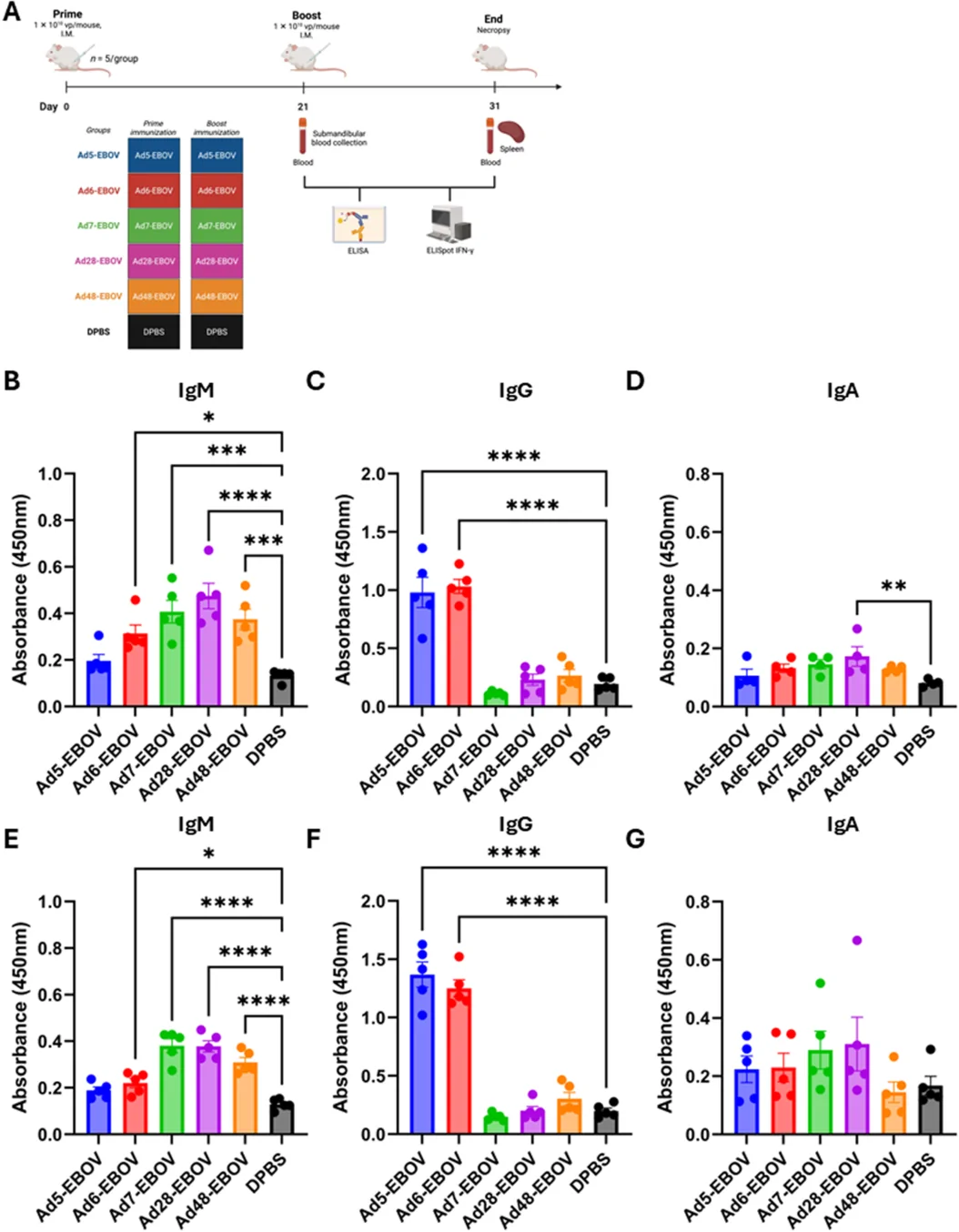
Single‐shot and homologous prime‐boost immune correlates. (A) Study design. Five mice per group were immunized intramuscularly with 10^10^ viral particles on Days 0 and 21. Blood and tissue collection occurred on Days 21 and 31, and serum antibody levels were measured by ELISA. (B) Single‐shot IgM. (C) Single‐shot IgG. (D) Single‐shot IgA. (E) Prime‐boost IgM. (F) Prime‐boost IgG. (G) Prime‐boost IgA. Data are expressed as mean with standard error (SEM). One‐way ANOVA with Dunnett's multiple comparisons used for statistical analysis. *p < 0.05, **p < 0.01, ***p < 0.001, ****p < 0.0001.

To assess the five Ad‐vectored EBOV GP vaccines, BALB/c mice (*n* = 5/group) were immunized intramuscularly with 1 × 10^10^ vp/mouse. Three weeks after immunization, a small amount of blood was collected by cheek bleed (Figure 2A) to assess antibody levels induced by a single‐shot vaccination. All vectors induced detectable GP‐specific IgM responses against the Ebola lysate following prime immunization. Compared to DPBS controls, IgM levels were significantly elevated in Ad6, Ad7, Ad28, and Ad48 groups, whereas Ad5‐induced IgM did not reach statistical significance (Figure 2B). Species C vectors (Ad5 and Ad6) demonstrated 5.1‐ to 5.3‐fold higher GP‐specific IgG responses, respectively, detected on Day 21 compared to the mock vaccine group. In contrast, species B (Ad7) and species D (Ad28, Ad48) vectors elicited comparatively lower IgG responses, which did not reach statistical significance compared to the DPBS control group (Figure 2C). Serum IgA levels were near the unvaccinated control levels for most of the vaccine groups, with fold changes between 1.3 and 1.8. Only the Ad28 vector reached statistical significance with a 2.1‐fold increase compared to the control group (Figure 2D).

### 3.3 Homologous Boosting Enhances Humoral Responses

Following the cheek bleeds, a homologous, intramuscular boost vaccine was administered on Day 21. Boost‐vaccinated mice were then sacrificed on Day 31 for terminal immune assessment (Figure 2A). All Ad vectors induced GP‐specific IgM responses after boost vaccination. IgM levels were statistically significant in the Ad6, Ad7, Ad28, and Ad48 groups when compared to the DPBS control group. The Ad5 vector again did not reach statistically significant IgM levels, suggesting the possibility of rapid antibody class switching (Figure 2E). Following boost vaccination, elevated IgG levels were produced across all vaccine groups compared to the unvaccinated control group. The Ad5 and Ad6 vectors in particular produced statistically significant IgG responses, with 6.8‐ and 6.3‐fold increases compared to the DPBS group (Figure 2F). Antigen‐specific serum IgA responses induced by vaccination were near baseline levels, with none of the vaccine groups achieving statistical significance (Figure 2G).

### 3.4 Heterologous Serotype Switching Enhances Humoral and Cellular Immunity Following Intramuscular Vaccination

**Figure 3:**
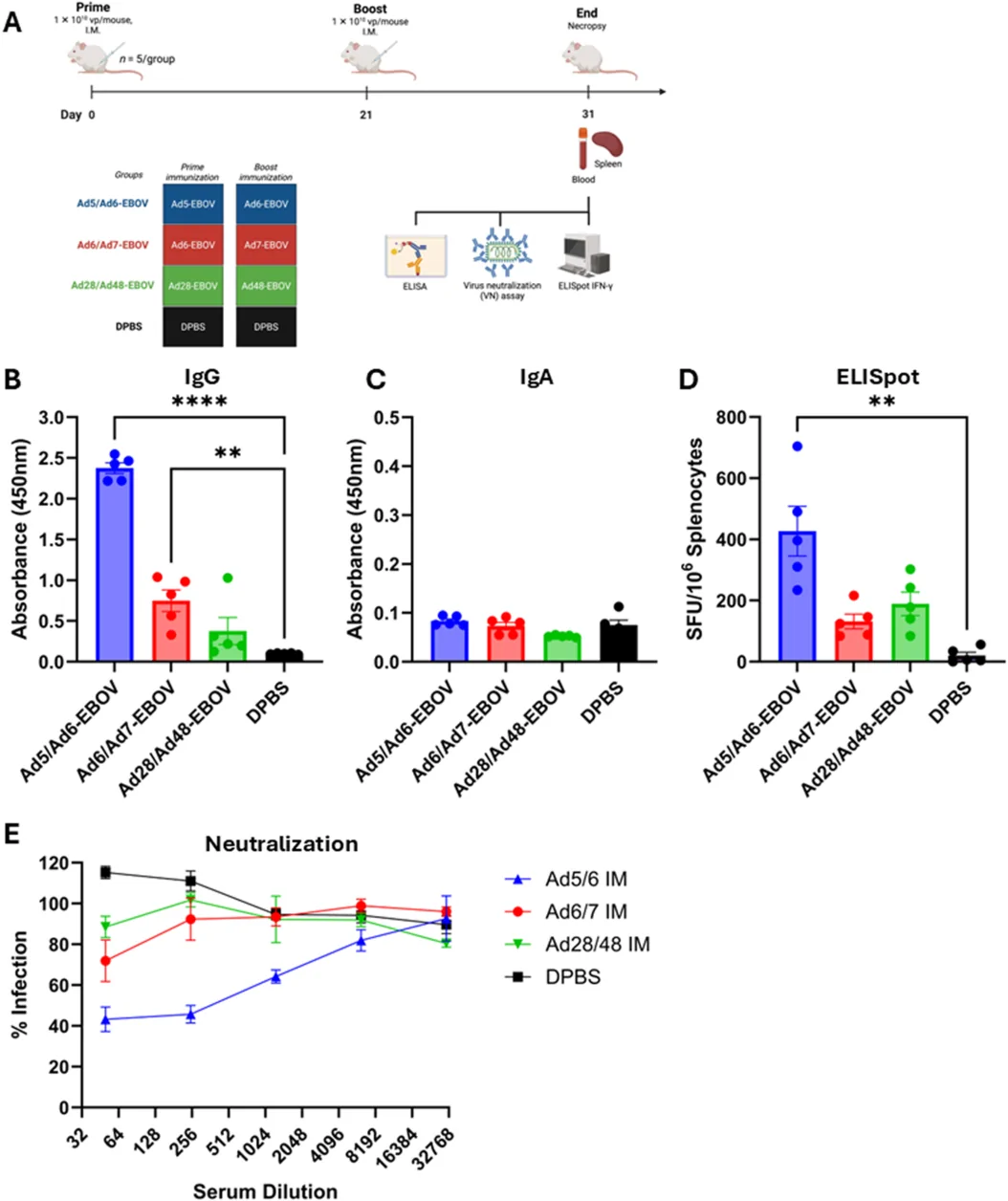
Serotype‐switched intramuscular vaccination immune correlates. (A) Study design. Five mice per group were immunized intramuscularly with 10^10^ viral particles on Days 0 and given a heterologous vaccination on Day 21. Blood and tissue collection occurred on Day 31. (B) Serum IgG measured by ELISA. (C) Serum IgA measured by ELISA. (D) IFN‐γ^+^ T‐cell measured by ELISpot assay. Results are reported as spot forming units (SFU) per million splenocytes. (E) Serum neutralization of Ebola pseudovirus. Data are expressed as mean with standard error (SEM). One‐way ANOVA with Dunnett's multiple comparisons was used for statistical analysis. **p < 0.01, ****p < 0.0001.

We next evaluated whether heterologous serotype switching could increase immune responses. Mice were primed intramuscularly with Ad5, Ad6, or Ad28 and boosted on Day 21 with Ad6, Ad7, or Ad48, respectively. Immune responses were subsequently assessed on Day 31 (Figure 3A). Serotype‐switched regimens significantly enhanced GP‐specific IgG responses compared to mock controls. The Ad5/Ad6 combination elicited the highest antibody levels, with a 24‐fold increase compared to the DPBS group. The Ad6/Ad7 group produced a 7.6‐fold increase, while the Ad28/Ad48 regimen produced a 3.8‐fold increase but did not reach statistical significance (Figure 3B). Consistent with intramuscular vaccine delivery, serum IgA responses remained near baseline levels across all groups (Figure 3C). Additionally, serotype‐switched, heterologous boosting resulted in measurable antigen‐specific cellular immunity. The Ad5/Ad6 regimen induced the strongest IFN‐γ T cell responses, with an average of 426.8 SFU/10^6^ splenocytes. The Ad28/Ad48 and Ad6/Ad7 regimens also generated antigen‐specific cell‐based immune responses (189.2 and 131.6 SFU/10^6^ splenocytes, respectively), although these were not statistically significant (Figure 3D). These data demonstrate that serotype switching enhances cellular immune induction relative to homologous boosting.

Functional antibody activity was evaluated using a pseudovirus neutralization assay. Sera from the Ad5/Ad6‐vaccinated mice demonstrated dose‐dependent inhibition of pseudovirus entry with a 50% inhibitory dose (ID_50_) titer of 369. Maximum neutralization activity was observed at the lowest tested dilution (1:50), which achieved a 56.8% decrease in pseudovirus infection (43.2% residual infection) compared to the virus‐only control. The Ad6/Ad7 and Ad28/Ad48 regimens exhibited comparatively lower levels of neutralization activity at a 1:50 dilution, with a 28.1% and 11.4% reduction in infection, respectively. None of the sera demonstrated any functional activity at the highest tested dilution (1:31,250). Additionally, sera from the mock vaccinated group showed no detectable neutralizing activity at any dilution (Figure 3E). Together, these findings indicate that Ad‐vectored heterologous serotype switching improves both the magnitude of T cell responses and functional antibody activity relative to the homologous prime boost regimen following intramuscular immunization.

### 3.5 Intranasal Serotype Switching Promotes Mucosal Immunity

**Figure 4:**
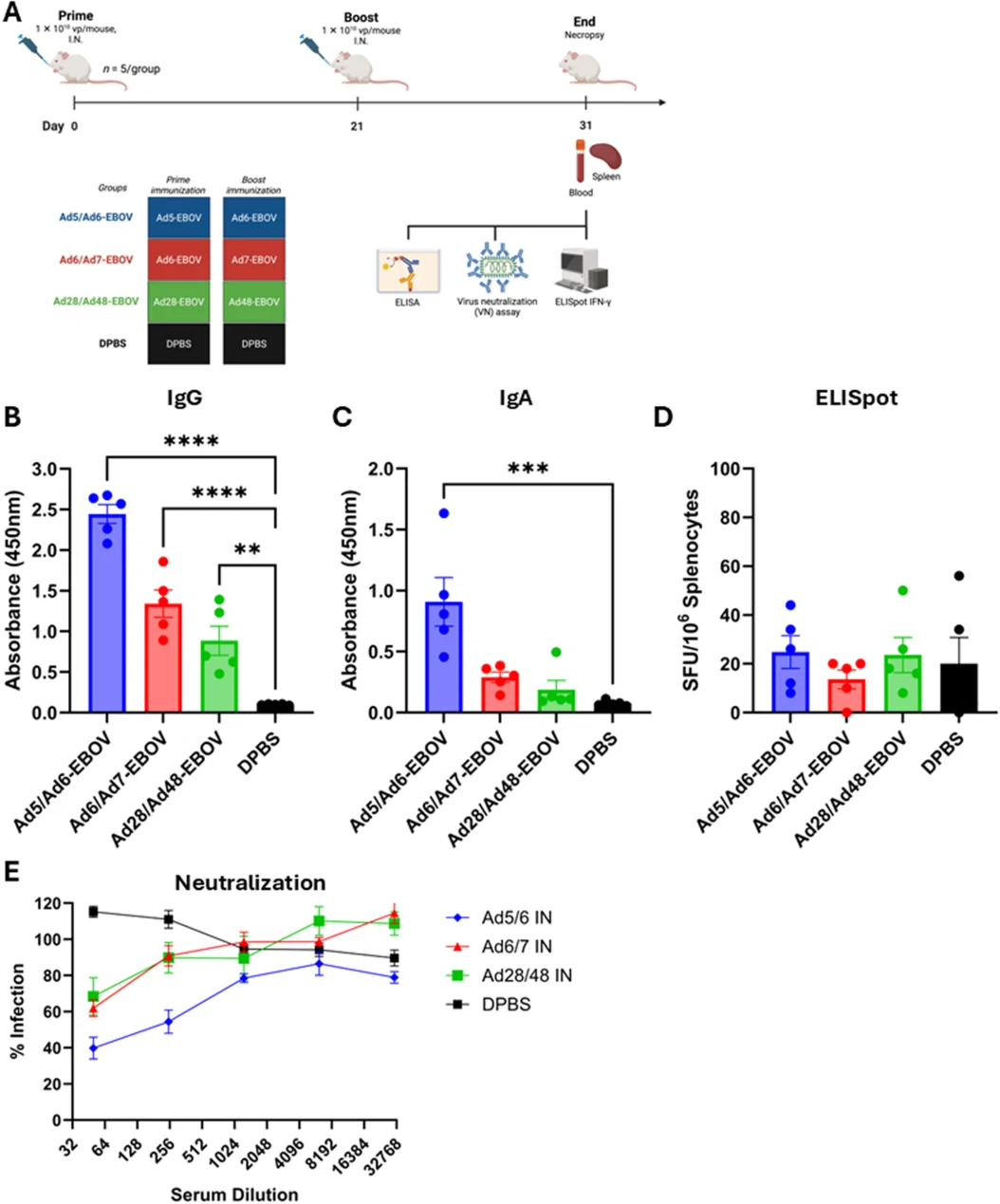
Serotype‐switched intranasal vaccination immune correlates. (A) Study design. Five mice per group were immunized intranasally with 10^10^ viral particles on Days 0 and given a heterologous vaccination on Day 21. Blood and tissue collection occurred on Day 31. (B) Serum IgG measured by ELISA. (C) Serum IgA measured by ELISA. (D) IFN‐γ^+^ T‐cell measured by ELISpot assay. Results are reported as spot forming units (SFU) per million splenocytes. (E) Serum neutralization of Ebola pseudovirus. Data are expressed as mean with standard error (SEM). One‐way ANOVA with Dunnett's multiple comparisons used for statistical analysis. **p < 0.01, ***p < 0.001, ****p < 0.0001.

To determine whether route of administration alters immune programming, mice were immunized intranasally using heterologous serotype‐switched regimens. Mice were primed with Ad5, Ad6, or Ad28 and boosted on Day 21 with Ad6, Ad7, or Ad48, respectively. Immune responses were then evaluated on Day 31 (Figure 4A). Intranasal vaccination induced robust GP‐specific IgG levels across all serotype‐switched groups, with the Ad5/Ad6 vaccine regimen eliciting a 24.7‐fold increase relative to the control group. Additionally, the Ad6/Ad7 group had a 13.5‐fold increase and the Ad28/Ad48 group had an 8.9‐fold increase (Figure 4B). Notably, intranasal delivery also induced serum IgA responses above background levels, particularly in the Ad5/Ad6 group, which had a statistically significant 12.1‐fold increase compared to the unvaccinated control group. The Ad6/Ad7 and Ad28/Ad48 groups also had increased IgA levels compared to the control group with a 3.8‐fold and 2.5‐fold increase, respectively (Figure 4C). In contrast to intramuscular vaccination, antigen‐specific IFN‐γ producing splenocytes were not detectable above background levels in any intranasally vaccinated groups (Figure 4D), suggesting that mucosal vaccine administration favors humoral immune responses over cellular immunity.

Sera from intranasally vaccinated mice demonstrated some ability to block EBOV pseudovirus entry when tested in a neutralization assay. The Ad5/Ad6 group had an ID_50_ titer of 194, with the 1:50 dilution reaching a 60.1% reduction of pseudovirus infection (39.9% residual infection) compared to the virus‐only control wells. Additionally, the Ad6/Ad7 and Ad28/Ad48 groups achieved 38% and 31.6% reduction of infection, respectively, at a 1:50 dilution. Mock‐vaccinated sera showed no inhibitory activity (Figure 4E). Together, these findings demonstrate that intranasal adenoviral vaccination induced systemic IgG and detectable IgA responses with functional neutralizing capacity but did not generate measurable splenic IFN‐γ T cell responses.

## 4 Discussion

The data presented here add to the growing body of evidence supporting adenovirus‐vectored vaccines as a promising platform for protection against Ebolavirus [30, 31, 32]. Recombinant adenoviruses are attractive vaccine vectors due to their capacity to induce robust and durable immune responses. Human adenoviruses comprise more than 80 recognized types distributed across seven species (A–G), with substantial diversity in receptor usage, tissue tropism, and global seroprevalence [33, 34]. This heterogeneity provides opportunities to optimize vaccine design by selecting vectors with distinct biological properties and by mitigating the impact of pre‐existing anti‐vector immunity. Here, we compared five human adenovirus‐vectored Ebola vaccines spanning across three species and evaluated their humoral and cellular immunogenicity in a murine model.

All five vectors elicited detectable antibody responses following prime immunization, which were further increased by booster vaccination. Among these, species C vectors (Ad5 and Ad6) induced the highest antibody titers as measured by ELISA. This enhanced immunogenicity observed with species C vectors may reflect differences in receptor usage and cell tropism. Species C adenoviruses utilize the Coxsackie–Adenovirus receptor (CAR), expressed in both mice and humans, whereas species B and D adenoviruses primarily utilize the more species‐specific desmoglein and CD46 receptors [35, 36, 37]. These findings support the importance of vector–host receptor compatibility in shaping vaccine immunogenicity in preclinical models [38].

We further examined heterologous prime–boost strategies through serotype switching, which has been demonstrated to improve immune responses [39]. In agreement with these studies, serotype switching in intramuscular regimens enhanced IFN‐γ^+^ splenocyte T cell responses relative to homologous prime‐boost vaccination. Although this work did not investigate secretion of other cytokines (e.g., IL‐2, TNF‐α, IL‐4, etc.) or various subpopulations of mucosal T cells induced by vaccination, the induction of systemic cellular immunity represents a critical component of vaccine‐mediated protection against EBOV infection [40]. Serotype‐switched regimens also generated higher antibody titers than homologous vaccination for both species C and D vectors, and sera from these animals demonstrated partial neutralizing activity in an in vitro pseudovirus assay. Together, these findings suggest that heterologous prime‐boost strategies may optimize both humoral and cellular immune responses in adenovirus‐based Ebola vaccines.

In addition to improving the durability and immunogenicity of ZEBOV vaccines, strategies to enhance vaccine accessibility and uptake remain a priority. Needle‐free delivery platforms may increase uptake, particularly in outbreak settings. We therefore evaluated intranasal administration as an alternative to intramuscular vaccination. Intranasal immunization elicited robust systemic antibody responses while also inducing antigen‐specific IgA, suggesting engagement of mucosal immune pathways. Studies have shown that Ebola virus survivors have early IgG responses and have suggested that IgA also contributes to protection [41, 42, 43, 44]. Notably, intranasal vaccination conferred levels of protection against ZEBOV pseudovirus entry that were comparable to intramuscular administration (ID_50_ titers of 194 and 369, respectively), although both of these values are high compared to other reported ZEBOV vaccines [45, 46]. Although the selected post‐primary and post‐boost sampling time points provide an assessment of established mucosal antibody responses, a more comprehensive time‐course analysis would better define the kinetics and durability of the antigen‐specific IgA response. Intranasal delivery did not elicit detectable IFN‐γ secreting splenocytes, which is common and not unexpected for this route of administration. It is possible that intranasal immunization induced lung‐resident memory T cell response or other localized lymphoid tissue responses [47]. Vaccination combinations such as IM prime followed by IN boost, or vice versa, may help to stimulate both systemic and mucosal humoral and cell‐based immune responses. Future studies should evaluate these vaccine candidates against infectious Ebola virus in vitro and in appropriate, outbred in vivo models, including both male and female non‐human primates, to determine whether the immunological differences observed here translate to meaningful differences in protective efficacy. Combinatorial strategies, such as pairing mucosal vaccination with platforms designed to enhance cellular immunity (e.g., transdermal or heterologous systemic boosting), may further improve overall protective responses.

Collectively, these results demonstrate that vector selection, serotype switching, and route of administration each substantially influence the magnitude and quality of immune responses elicited by adenovirus‐vectored Ebola vaccines. Strategic integration of these parameters could help to improve upon existing Ebola vaccines by providing insights into improving immunogenicity, flexibility, and translational potential of the next‐generation of Ebola vaccines.

## Author Contributions

Conceptualization: Eric Weaver. Formal analysis: Joshua Wiggins, Adthakorn Madapong. Funding acquisition: Eric Weaver. Investigation: Joshua Wiggins, Adthakorn Madapong. Methodology: Joshua Wiggins, Eric Weaver. Project administration: Eric Weaver. Resources: Eric Weaver. Supervision: Eric Weaver. Validation: Joshua Wiggins, Eric Weaver. Visualization: Joshua Wiggins, Adthakorn Madapong, Eric Weaver. Writing – original draft: Joshua Wiggins. Writing – review and editing: Joshua Wiggins, Adthakorn Madapong, Eric Weaver. All authors have read and agree to the final version of the manuscript.

## Conflicts of Interest

The authors declare no conflicts of interest.

## Supporting information


Supporting File

## Data Availability

All data published in this study will be made available by the corresponding author upon request.
